# The Working Hours of the Private Nurse: A Problem to Be Solved

**Published:** 1919-05-17

**Authors:** F. A. Sheldon

**Affiliations:** Lady Superintendent, Guy's Hospital Trained Nurses' Institution, S.E. 1.


*To the Editor of* The Hospital.

### The Working Hours of the Private Nurse: A Problem to be Solved.

*To the Editor of* The Hospital.

Sir,—For some little time the medical profession and the public in general have interested themselves in the working hours of the hospital probationer, staff nurse, and sister. I should very much like to see the question of the hours on duty of the private nurse treated in a sympathetic and imaginative manner in the medical and daily Press. The abnormal difficulties of war-time food and coal shortage and the entire absence of domestic help need not be touched upon, or the terrible demands of the influenza epidemics. A solution, which will be far from easy, is wanted to meet the everyday conditions now before us.

The chief obstacles which, so far, have prevented the granting of an eight-hour day or night in hospitals are :—1. Lack of funds to salary and maintain additional staff. 2. Lack of accommodation for the same. 3. And the opinion, in many minds, of the undesirability of treatment being carried out by three sets of nurses. Now all these difficulties are accentuated in the ordinary home. The fees, board and laundry, and travelling expenses of the one nurse are often a serious expense, a still greater difficulty to double, and few could treble them. Sleeping accommodation in most houses and all flats is limited. The Box and Cox system is uncomfortable and would not be tolerated in hospital nurses' homes, and where, except in a palatial residence, is a resting-place for the third nurse to be found? Most important of all, how is the treatment and care of the patient to be safeguarded with even the most helpful relatives at hand? There is a general tendency to think that, if for the time being the nurse is working at high pressure with an acute case, her next will be a light one, or she can take a short holiday before starting work again; also that attendance upon one patient cannot possibly be as arduous as nursing in a ward. With numbers there is always, however, encouragement, and in the presence of fellow-workers an incentive. A busy hospital day may produce physical exhaustion, but not, I think, to be compared to the exhaustion caused by the mental strain of spending a long night alone in one room with a serious pneumonia or heart case.

Surely more might be done in employing a nurse to relieve the nurse in residence, engaged perhaps from a local nursing home, or a doctor could permanently retain in his service, living in his neighbourhood, a "relieving" nurse, he, of course, charging his patients for her visits, which could be by the hour. The work of the private nurse is of immense value to the community, and from its very nature can never entirely be bound by rules. She would be the first to acknowledge that it is a life of great opportunity and that she meets many good friends and much kindness. Some share, however, should be hers in the general rearrangement of conditions of service and opportunities for leisure, as in all other lives. What solution have your readers, doctors, and patients to offer of the problem of the private nurse's working hours?—Yours faithfully,

F. A. Sheldon,
Lady Superintendent, Guy's Hospital Trained Nurses' Institution, S.E. 1.

May 12, 1919.

